# Quality of reporting inflammatory bowel disease randomised controlled trials: a systematic review

**DOI:** 10.1136/bmjgast-2023-001337

**Published:** 2024-04-17

**Authors:** Morris Gordon, Jamal Khudr, Vassiliki Sinopoulou, Svetlana Lakunina, Aditi Rane, Anthony Akobeng

**Affiliations:** 1 School of Medicine, University of Central Lancashire, Preston, UK; 2 Blackpool Families Division, Blackpool Victoria Hospital, Blackpool, UK; 3 Plastic Surgery, Manchester University NHS Foundation Trust, Manchester, UK; 4 Gastroenterology, Sidra Medicine, Doha, Ad Dawhah, Qatar; 5 School of Medicine, Weill Cornell Medical College-Qatar, Doha, Qatar

**Keywords:** CLINICAL TRIALS, IBD, CROHN'S DISEASE, ULCERATIVE COLITIS

## Abstract

**Objective:**

Our objective was to perform a systemic evaluation of the risk of bias in randomised controlled trial (RCT) reports published on inflammatory bowel disease (IBD).

**Design:**

We assessed the risk of bias using the Cochrane tool, as indicators of poor methodology or subsequently poor reporting. We systematically selected, with dual independent judgements, all studies published on IBD with no time limits and assessed the methodological quality of included studies again using independent dual ratings.

**Results:**

563 full texts were included after selection and review. No abstract publications were free of any source of bias. Full-text publications still fared badly, as only 103 full-text papers exhibited a low risk of bias in all reporting domains when excluding blinding. RCTs published in journals with higher impact factor (IF) were associated with an overall reduced rate of being at high risk. However, only 6% of full RCT publications in journals with an IF greater than 10, published in the past 5 years, were free of bias.

The trend over time is towards improved reporting in all areas. Trials published by larger author teams, in full-text form and by industry and public sponsorship were positively correlated with a lower risk of bias. Only allocation concealment showed a statistically significant improvement with time (p=0.037).

**Conclusion:**

These findings are consistent with those of other specialties in the literature. While this unclear risk of bias may represent poor reporting of methods instead of poor methodological quality, it leaves readers and future secondary researchers with significant questions regarding such key issues.

**WHAT IS ALREADY KNOWN ON THIS TOPIC**

- All randomised trials published should report using the Consolidated Standards of Reporting Trials guidance, released in 1995. This is more important now as the use of Grading of Recommendations, Assessment, Development and Evaluations (GRADE) as a system to support guideline decision-making means poor method reporting of individual studies impacts the certainty of overall outcomes used to make recommendations.

**WHAT THIS STUDY ADDS**

- When comparing studies published in the past 5 years in inflammatory bowel disease (IBD) to those before, statistically significant improvement was only noted in one out of the six risk of bias domains. The highest impact journals in the last 5 years still publish less than 1 in 10 IBD trials at low risk of bias in all areas.

**HOW THIS STUDY MIGHT AFFECT RESEARCH, PRACTICE OR POLICY**

- Reporting of risk of bias must be a priority for all researchers and peer reviewers as poor reporting threatens the certainty of all decision-making by guidelines committees when using the GRADE framework to make decisions internationally. Journals should strongly endorse adherence to reporting guidelines to authors and highlight these six key criteria as mandatory for clear reporting for all randomised controlled trials.

## Introduction

 Randomised controlled trials (RCTs) are the study of choice for evaluating the efficacy of interventions in the management of inflammatory bowel disease (IBD).^1 2^ Their methodological approach to reduce the risk of bias (RoB) ensures that the true effects of the intervention are reported in a manner that can best represent clinical reality.

In considering published RCTs, it is difficult to distinguish poor writing from poor research quality. All studies should report using the Consolidated Standards of Reporting Trials (CONSORT) statement’s minimum methodological standards of reporting that were initially released in 1995.^3^ While the CONSORT statement is a requirement for major journals, especially those with the top 1% of impact factors (IF), many journals do not mandate this which often leaves readers with uncertain interpretations. While peer review can and should address this and be expected to adhere to a three-decade-old standard, there are long-standing concerns with the validity and reliability of this process within the field of academia.^3 4^

Quality is a sometimes abstract concept, often a subjectively understood term. It has been further complicated in the last decade with the hugely significant emergence of a consensus on rating the quality of a whole evidence base for each outcome through the use of Grading of Recommendations, Assessment, Development and Evaluations (GRADE).^5^ For individual RCTs, quality is typically described as ‘design and RCT conduct, to prevent systematic errors, or bias’.^6^ Bias occurs when the results of a study do not represent the truth because of the inherent limitations in the design or conduct of a study.^7^

The Cochrane RoB tool is employed to judge key elements within evidence reviews.^8 9^ Selection bias, which includes appropriate randomisation, also includes the difficult-to-understand principle of allocation concealment (AC) which leads to overestimates of the treatment effect of close to 40% if poorly reported.^10^ This is best understood with an example. In an RCT comparing biological IBD treatment to a placebo, even with an appropriate randomisation schedule, if the allocation schedule is available during recruitment, a researcher aware that the next patient will receive a biologic might avoid a patient with difficult venous access. This can create an imbalance between groups. This is not the same as blinding, which can be impractical, but concealing allocation schedules, which is always feasible and crucial to avoid bias. Other sources of bias do exist that are not specifically accounted for within the Cochrane criteria and are usually accounted for and identified through overall assessments of available data. The other bias (OB) section within the Cochrane criteria allows for documentation of the above concerns; however, the other six criteria have universal agreement as the most specific sources of bias with the highest influence on reported outcomes.

A previous study on over 20 000 RCTs in Cochrane reviews until 2014 noted improved reporting trends in all these key items of reporting within studies published between 1997 and 2008.^11^ In IBD, a limited evidence base informs the treatment of vast patient numbers. Recently, most IBD international guideline committees have adopted the GRADE approach for quality and recommendation assessments. Imperatively, GRADE evaluates bias risk at the outcome level, not per study. Thus, despite the presence of some studies at low RoB, overall evidence quality can be downgraded if other studies exhibit higher risk.^5^ This emphasises the increasing need for unbiased RCT reporting to distinguish genuine quality concerns from reporting issues.

We set out to examine how these key elements of RoB are reported within IBD RCTs and factors associated with a higher RoB through a systematic review of all IBD RCTs published since the CONSORT statement (1995).^3^ This will be completed using the Cochrane RoB tool to a whole sample of published RCTs.

## Materials and methods

The protocol for the review has been uploaded to a repository (Repository ID: 33117).^12^ An ethical screening tool was completed, and full ethical approval was not required.

### Study selection

We performed a systematic electronic database search of all the RCTs published on IBD from the following databases: MEDLINE, Embase, CENTRAL and The Cochrane Register (online supplemental appendix 1). All studies on IBD were collected up to the date of the search (September 2020). All citations were imported into Rayyan and deduplicated for abstract screening in duplicate at the title and abstract level.

Inclusion criteria included RCTs involving patients with either Crohn’s disease, ulcerative colitis or a combination of the conditions. Trials including all age groups and patients in any disease state were considered. Studies could involve any form of intervention compared with any other intervention, placebo, no treatment or usual care. Studies could include any outcome measures. Studies could include any outcome measures. All early phase II and phase III trials were included. Phase I, animal trials, quasirandomised trials and other study types were not included. No time or language restrictions were imposed.

Three authors (JK, VS and SL) independently reviewed all titles and abstracts, with two author judgements required before the progression of a paper to full-text assessment. Any disagreements were resolved by a fourth author (MG).

All journal articles chosen for full-text review were evaluated independently again by two authors (JK and AR) to assess inclusion criteria to consider for analysis, with the third and fourth authors (MG and VS) resolving differences. RCTs published in abstract form only, which met the eligibility criteria, were also included in this review.

### Data extraction and quality assessment

From included RCTs (full text and abstract), data were simultaneously extracted independently by the above authors (JK and AR), and using a standardised form, an RoB judgement was recorded for each item, as discussed above. Additionally, key demographic and descriptive data were extracted, including the publication type (full text/abstract/letter), year of publication, journal source, language, number of authors, funding source and number of study centres. Disagreements were once again resolved through the involvement of a third author (VS and MG).

A matching algorithm and manual validation were used to standardise journal names and eliminate abbreviations. We visited the website of each listed journal, if available, and extracted its up-to-date IF. The IF centile and year-specific IF for each publication were then collected from Journal Citation Reports.

The publication characteristics were then combined with the collected RoB assessment for each RCT, and as such were categorised as ‘low’, ‘high’ or ‘unclear’ for each key RoB item as per the Cochrane RoB tool.^6^ In the context of missing information, primary authors were not contacted.

### Assessment of methodological quality and conduct rigour

Our assessment of RCTs’ methodological quality was based on the premise that a key item is considered at unclear RoB when inadequate information is provided to allow judgement of high or low risk, in line with the tool.^6^

### Analysis

The data were first analysed to assess the RoB for each key methodological item (low, high, unclear). We initially considered the whole sample of trials. Then, we compared the full text to the abstract overall. For subsequent analyses, abstracts were removed to avoid conflating limited reporting space with method quality.

For the full RCT publications, we then evaluated each criterion to determine the proportion of trials at low and high RoB. This was then used to determine the evolution of poor reporting over time, its association to IF, IF date, centile of IF, funding source, reported form, study centre and the number of authors. The synthesis was narrative and descriptive, in line with appropriate reporting guidance.^13^ We reported the overall proportions of key items using both bar graphs and pie charts and then explored the impact of other factors on these key items as above.

We calculated using SPSS (v27) descriptive statistics and conducted χ^2^ tests for statistical differences as per online supplemental appendix 2.^14^ Comparison was made between the group of papers that had a low RoB to those with high and unclear RoB judgements as markers of poor reporting.

## Results

### Selection process

**Figure 1:**
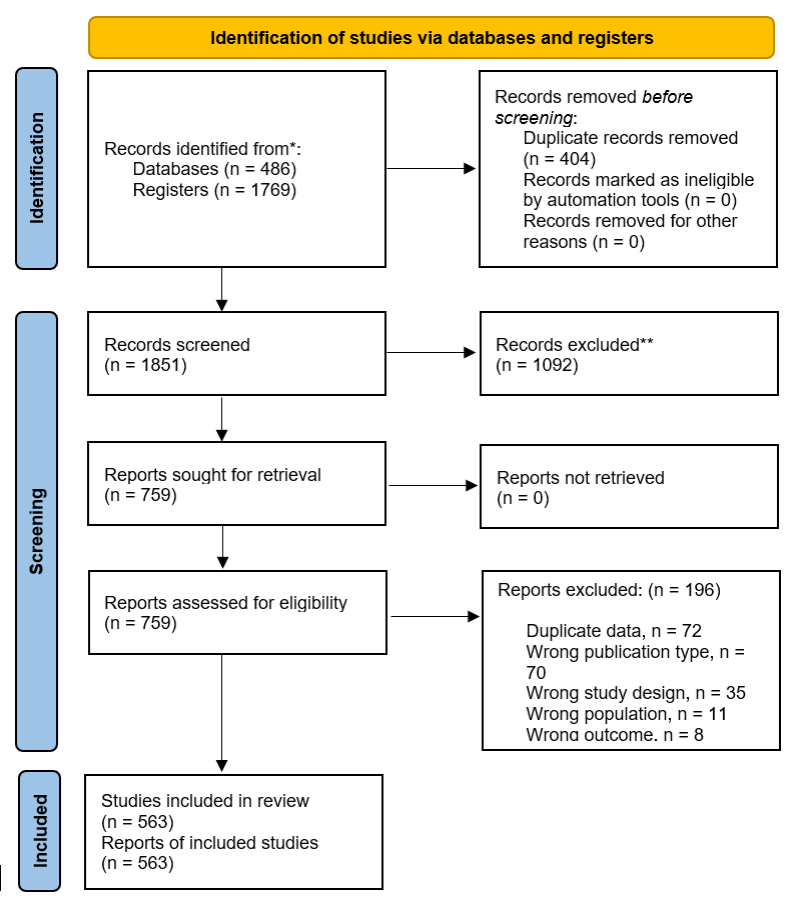
Preferred Reporting Items for Systematic Reviews and Meta-Analyses (PRISMA) flow diagram outlining the screening process. *Totalled from different sources in each category. **All with indepdent dual decision making, no use of Artifical Intelligence tools

After excluding duplicates, 1851 unique papers were examined and published in 167 journals (figure 1). After abstract and full-text screening, 563 RCTs were included, encompassing 362 unique full-text articles (median year of publication 2015, IQR 2006–2018) reported in 86 journals (table 1).

**Table 1:** Characteristics of included RCTs

Characteristic	n (%) unless otherwise specified
Publication year	2015 (2006–2018), 1974–2020
Published in journal with impact factor	549 (97.5)
Journal impact factor*[Table-fn T1_FN1]	
≥10	285
5–10	143
<5	121
Published in journal without an impact factor	14
10 highest represented journals	
* Gastroenterology*	133 (23.6)
* Journal of Crohn’s and Colitis*	65 (11.5)
* Gut*	50 (8.9)
* Inflammatory Bowel Diseases*	36 (6.4)
* American Journal of Gastroenterology*	32 (5.7)
* Alimentary Pharmacology & Therapeutics*	25 (4.4)
* United European Gastroenterology Journal*	24 (4.3)
* New England Journal of Medicine*	21 (3.7)
* Clinical Gastroenterology and Hepatology*	15 (2.7)
* Lancet*	13 (2.3)

* Median (IQR), min-max.

RCT, randomised controlled trial.

### General characteristics

The IF for the journals can be seen in table 1. RCTs were most likely to be reported in journals with IF ≥10 (285 (50.6%)). A large majority were written in English. Of the RCTs included, 14 were published in journals without an IF (2.5%).

### Methodological quality assessment

#### Overall assessment

**Figure 2:**
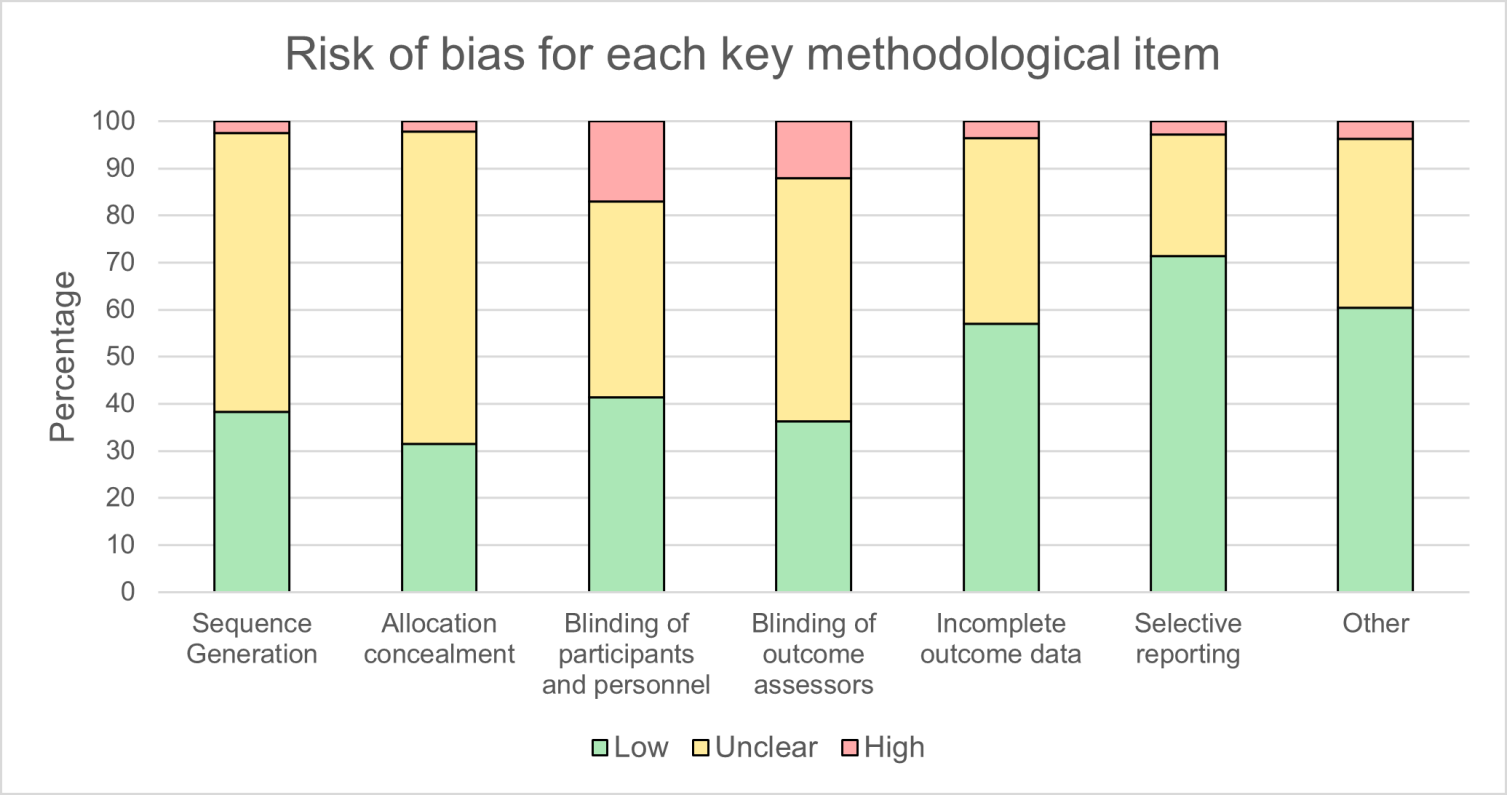
Risk of bias for included elements.

For sequence generation (SG) and AC, the proportion of trials judged as ‘low risk’ and ‘unclear risk’ was 59.1% and 66.4%, respectively. The unclear RoB was similar for the blinding of participants and personnel (BPP) at 41.6% and the blinding of outcome assessors (BOA) at 51.7%. For incomplete outcome data (IOD), selective reporting (SR) and OB, the figures were 39.4%, 25.8% and 35.9%, respectively (figure 2).

The proportion of trials at ‘high risk’ of bias of all trials was 2.5% for SG and 2.1% for AC. It was significantly worse for the components of blinding where it was at 17.1% for BPP and 12.1% for BOA. The proportion of studies at ‘high risk’ for IOD, SR and OB was 3.6%, 2.8% and 3.7%, respectively (figure 2).

#### Abstract-only versus full-text publications

**Figure 3:**
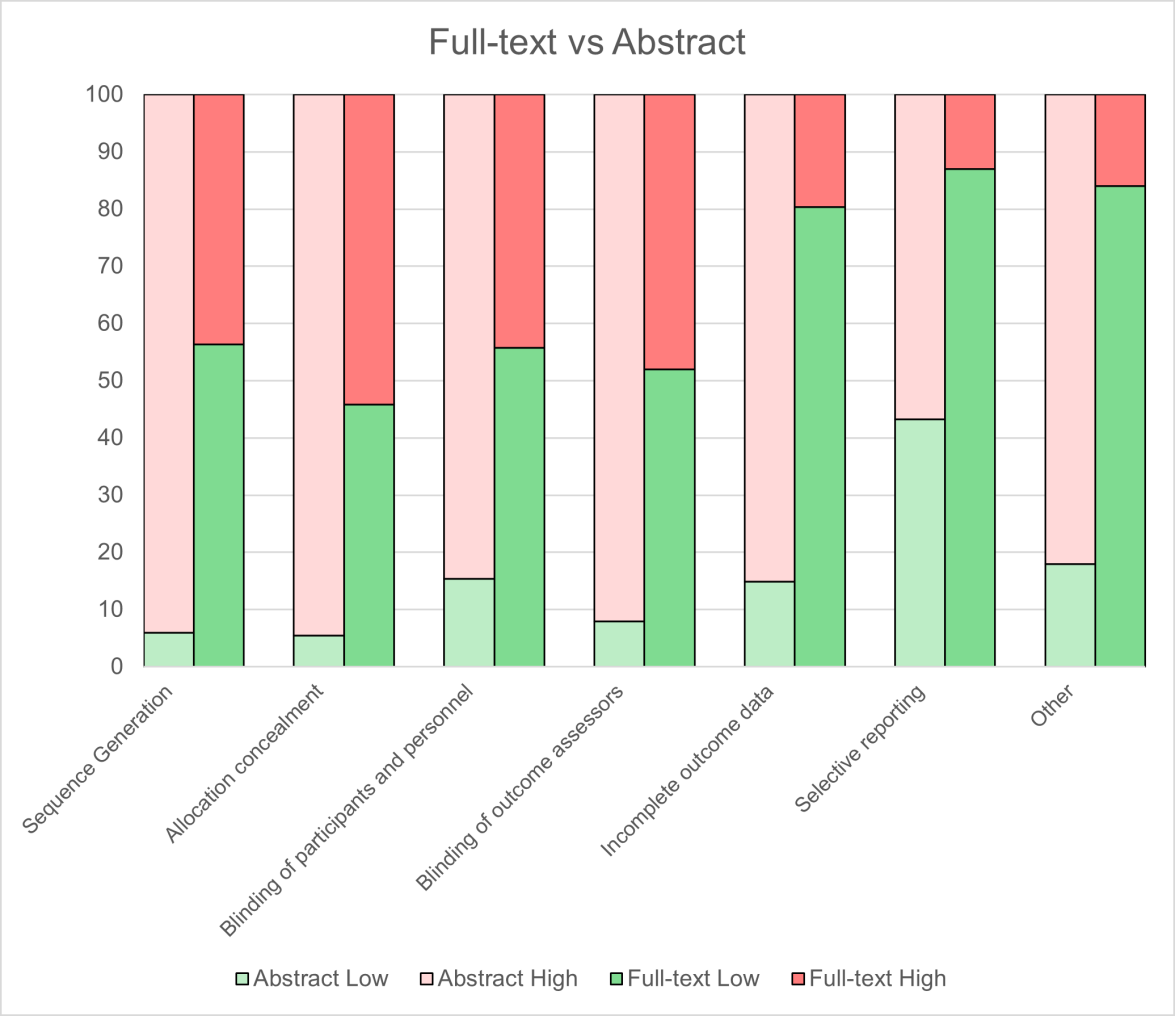
Risk of bias comparing full-text to abstract-only studies.

Trials published in full-text form compared with those in abstract form had a reduced proportion of ‘unclear risk’ of bias for all six RoB items. When considering unclear RoB as a surrogate for poor reporting, and consequently poor methodology, significant disparities can be seen between full-text and abstract-form publications when assessing risk (figure 3). This was particularly evident for IOD data which experienced a 65% increase in high and unclear RoB when reported in abstract form.

All subsequent analysis is presented with RCTs published in abstract form removed from the sample.

#### Author team size

**Figure 4:**
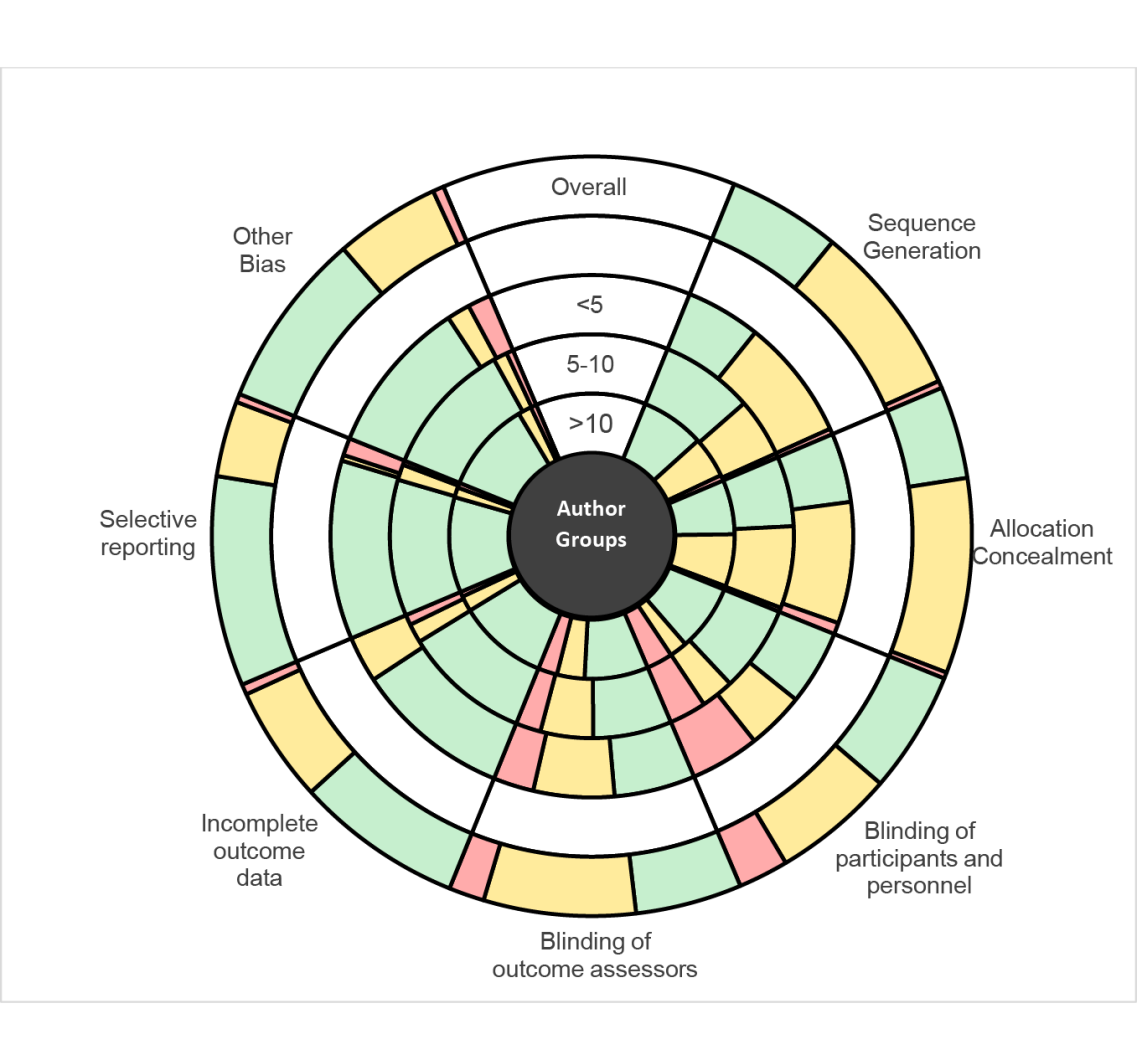
Risk of bias comparing author team size.

When comparing author groups with fewer than five authors to those with more, there were notable differences in the quality of trials (figure 4). Specifically, trials conducted by teams with less than five authors showed a significant increase in ‘unclear risk’ regarding SG and BPP. The increase was 35% (p=0.014) for SG and 19.9% (p=0.021) for blinding, indicating a higher proportion of unclear risk in smaller author teams.

#### Impact factor

**Figure 5:**
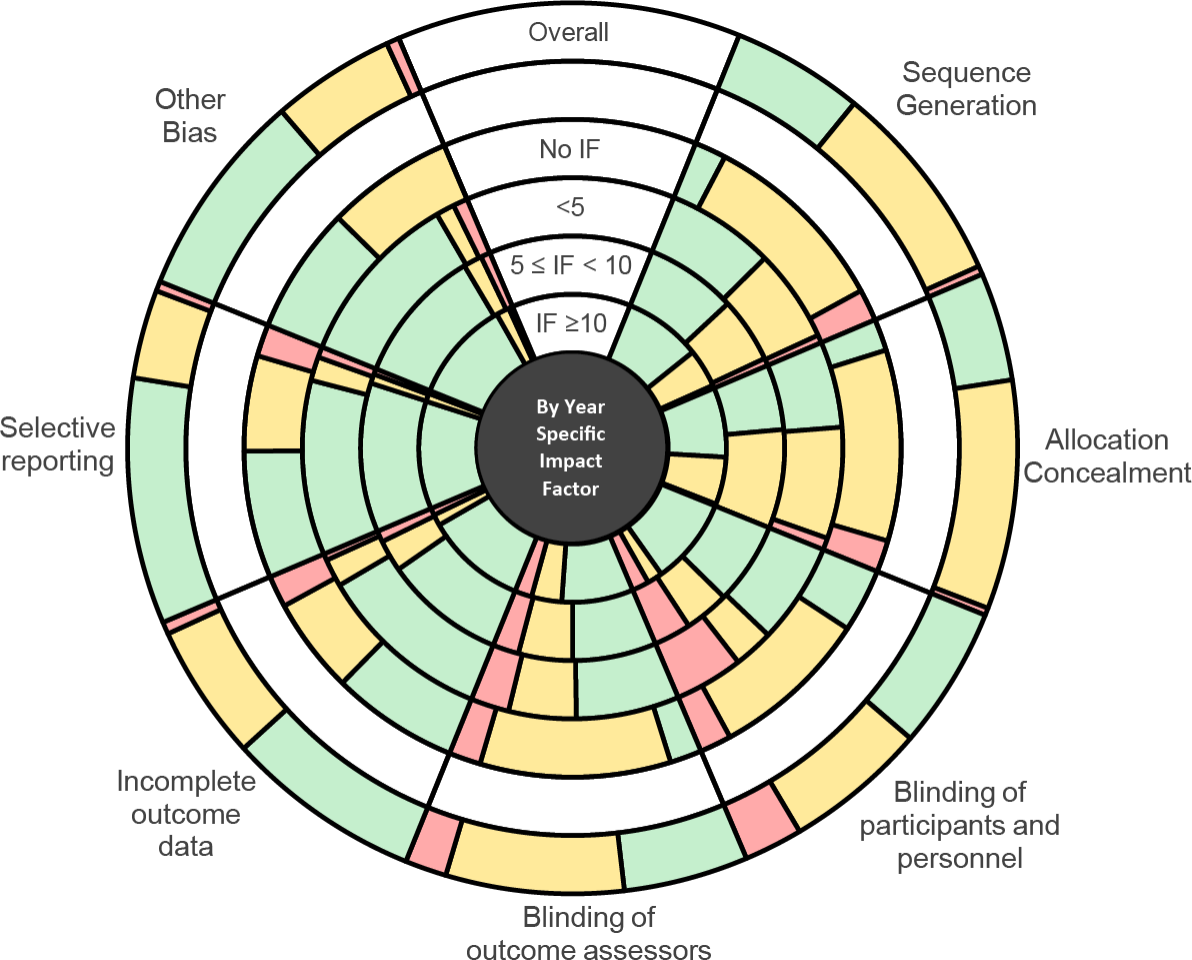
Risk of bias comparing journal impact factor (IF) of published studies.

Journals with high IF were associated with a lower proportion of trials at ‘high’ or ‘unclear risk’ compared with those with low or no IF (figure 5).

Statistical differences were seen in five out of the seven RoB components when comparing RCTs published in journals with an IF compared with those without one. Similar trends were evident between RCTs published in journals with an IF <5 to those >5. No statistically significant differences were evident when comparing journals with an IF between 5 and 10 to those with an IF >10.

This trend was evident when journals were examined by their IF centile, as trials with no IF were 40.4% more likely to have unclear or high judgements compared with those above the 90th centile.

#### Funding source

**Figure 6:**
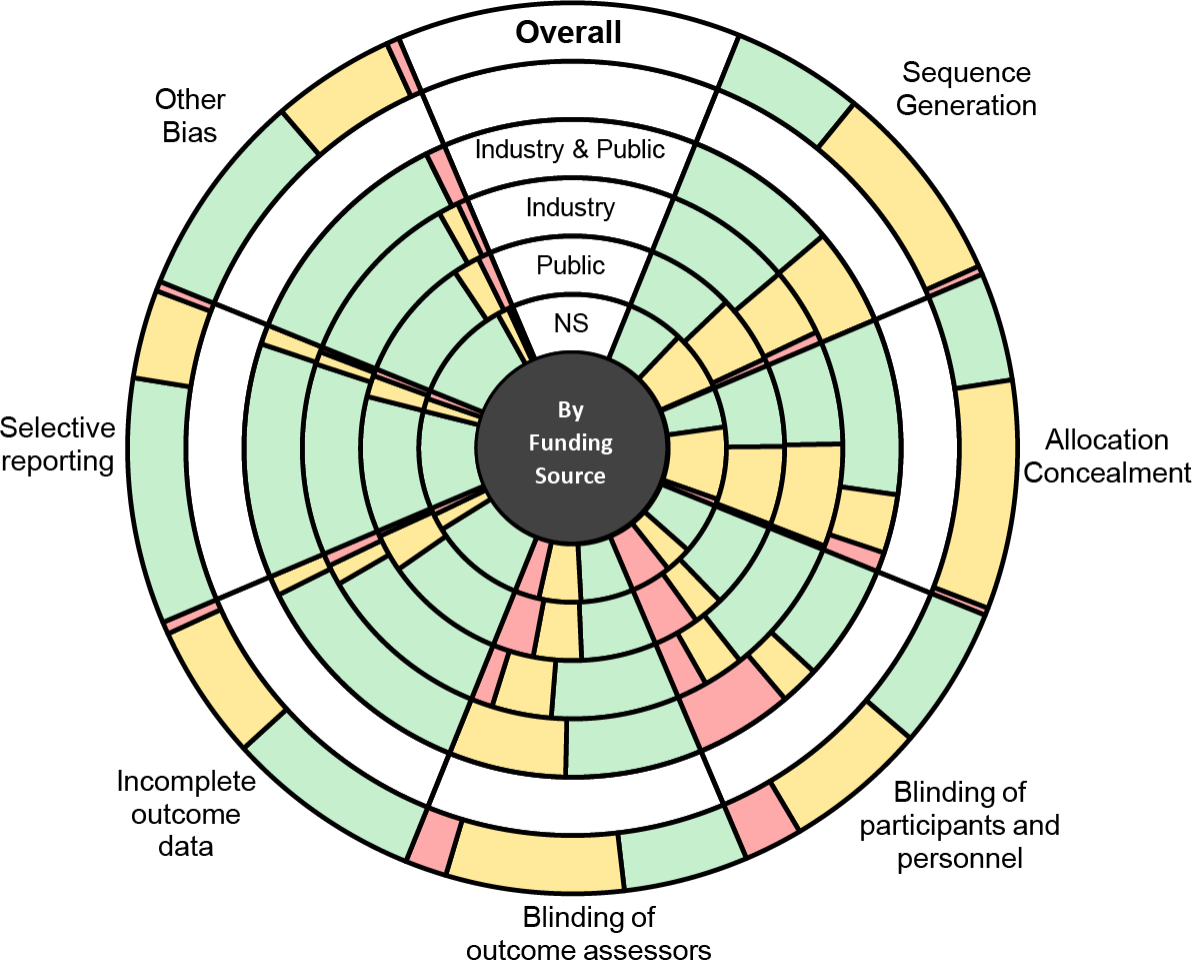
Risk of bias comparing funding source and methodological quality. NS, not specified.

We found a clear difference in the proportion of trials at unclear risk by funding source (figure 6). Studies without a funding source or those that did not specify any were more likely to have a ‘high’ or ‘unclear’ RoB when compared with funded studies, especially when considering AC (p=0.0029) and BPP (p=0.0034). Public sponsorship-funded studies experienced a higher proportion of trials at ‘unclear’ and ‘high risk’, in comparison to studies with industry funding, when considering BOA (p=0.0187), IOD (p=0.0425) and SR (p=0.0315). No statistically significant difference was noted between industry and public sponsorship-funded studies when compared with those funded by the industry.

#### Multicentre versus single-centre trials

Studies conducted in a multicentre setting conveyed lower RoB in all methodological items compared with studies conducted at multiple centres (online supplemental appendix 3). For example, when looking at AC it is evident that 49.7% of trials conducted in single centres were at ‘unclear risk’ of bias compared with 33.8% of those in a single centre.

### Evolution of the quality of RCT reporting

The proportion of RCTs at unclear RoB displayed mixed results. For AC, SG, BPP and BOA, they experienced a fall of 44%, 27% and 47%, respectively, in the ‘unclear risk’ categories when comparing data in RCTs from <1985 to those from 2016 to 2020 (online supplemental appendix 4). This is then contrasted by IOD, SR and OB which experienced a corresponding 7%, 2% and 6% increase in the proportion of trials at ‘unclear bias’ when examining RCTs published between the same periods.

Journal IF was not significant in the evolution of unclear RoB over time as similar inconclusive data were prevalent when comparing journals with different IF.

It is worth noting that when comparing the output from the most recent 3 years, when comparing the risk of ‘unclear’ or ‘high’ bias between studies published after 2017 to those prior, only AC showed a statistically significant improvement with time (p=0.037).

When blinding is excluded from the assessment tool due to its impracticality in specific study types, like surgical trials where attaining a low-risk status is pragmatically unattainable, we found that 103 (18.29%) papers exhibited a low RoB in all reporting domains. All of these low-risk papers were published in full-text form. When examining the breakdown between IF categories, 36 (6.3%) were published in journals with IF <5, 25 (4.4%) with IF 5–10 and 42 (7.5%) with IF >10. When examining the 127 papers that were published in the last 5 years in journals with an IF >10, eight trials (6.3%) were free of bias in all domains (excluding blinding).

### Χ^2^ evaluation of predetermined factors

There were statistically significant differences in reporting in journals with an IF compared with those without one when looking at all RoB categories except for AC and BPP (online supplemental appendix 5). Similarly, there were statistically higher chances of the studies being at low RoB when comparing studies published in journals with an IF >5 to those published in journals with an IF <5. In the final analysis, no statistically significant differences were present when comparing studies published in journals with an IF between 5 and 10 to those with an IF >10.

When considering funding, it is evident that having a funding source is associated with a statistically significant improvement in reporting AC and BPP. Having an industry source of funding demonstrates statistical improvement in BOA, IOD and SR when compared with studies funded by public sponsorship. Studies funded by both industry and public sponsorship sources had no differences when compared with those that were only funded by industry sources.

Looking at reporting over time, studies published in the last 3 years only exhibited a statistically significant improvement in AC when compared with those published before 2017.

## Discussion

This review indicates that, despite reporting improvements over time, many full-text published trials still are not at low RoB in essential reporting elements.

Our study revealed lower RoB levels when abstracts were excluded. This highlights concerns for conference organisers, guideline developers and others who rely on abstracts. Often, abstracts are the only trial form for years, sometimes never becoming full papers, impacting future GRADE-based decisions. This poses a question about the use of abstracts in systematic reviews due to potential bias. With almost a third of RCTs remaining unpublished after abstract presentation, relying on this ‘grey’ literature introduces publication bias favouring positive results.^15 16^ To mitigate this bias, more thorough peer reviews for conference abstracts and a study design registry or detailed display tool for abstracts are essential.

Within the full-text trials only, the same patterns were seen with improvements over time, but low-risk reporting in all categories is still a rarity. This has huge consequences as it directly lowers the strength of the recommendations during GRADE guideline development which is paramount.

Our study and those in the literature in other fields have shown that high rates of uncertainty exist in assessing the RoB, particularly for the SG and AC components.^17^ Studies reporting unclear or high risk of selection bias were more likely to reach positive conclusions and exaggerate intervention effects by an average of 9%.^17 18^ While the complete elimination of high RoB may not always be achievable due to the specific constraints of certain study designs, a significant portion of the unclear RoB can be attributed to inadequate reporting practices. It is critical to recognise that unclear RoB, being largely preventable, is unacceptable as it substantially limits the interpretation, application and certainty of available data. Additionally, while cautious interpretation of unclear RoB data by readers is essential, the implementation of the GRADE approach, which prioritises overall outcomes over individual study evaluations, typically leads to more guarded recommendations in evidence-based guidelines.^5^ This consideration gains further importance in light of the increasing integration of the GRADE methodology on an international scale, particularly in the UK, as evidenced by the forthcoming British Society of Gastroenterology guidelines.^19^

While the lack of blinding influence on effect measures has been highlighted,^17^ its challenging implementation in certain trials, such as surgery, emphasises the significance of AC for mitigating selection bias, given its feasibility and cost-effectiveness. While some trials suggest an overestimation of treatment effects due to inadequate AC,^1020^,^22^ others do not.^23^,^27^ Regardless, instances of insufficient AC reporting should be reduced.

Industry studies often show biased outcomes.^28^ Industry-backed studies tend to overstate efficacy, while non-industry ones more frequently report harmful effects.^29 30^ Industry funding also correlates with a higher bias risk in SG than public sponsorship.^31^ In our dataset, industry-funded studies reported a ‘low risk’ of bias for key items when compared with publicly sponsored ones, possibly reflecting their understanding of these key items’ significance. Other explanations might be their correlation with other factors that improve the RoB reporting such as larger author groups, multicentre settings and publishing in higher IF journals.

The evolution of reporting has been shown to improve over time throughout the literature and across different specialties and could be linked to the development of reporting guidelines, especially CONSORT.^32^,^34^ With a prominent upswing in reporting quality at the point of the introduction of the CONSORT statement in 1995 in our data (online supplemental appendix 4), a generalised improvement in reporting methodology is quite discernible over the 5 years after the release of the statement. Examining the data, it is interesting to note that the percentage of poorer quality papers has primarily remained constant over time since then and it is unclear why this progress has stagnated.

Our findings demonstrate that studies with a lower RoB tend to be published in high-impact journals, consistent with prior research.^35^ Whether attributed to improved funding, abundant resources or larger samples remains undetermined. Notably, even recent cohorts in these highest impact journals were still rarely at low RoB in all areas, highlighting a missed opportunity for these highly cited journals to champion transparent and CONSORT-aligned IBD reporting. It is unclear why this is the case.

We further recommend that journals strongly endorse the adherence of reporting guidelines to RCT authors and highlight these six key criteria as mandatory for all RCTs among peer reviews.

This study’s limitations include its focus on IBD RCTs, potentially limiting generalisability to other specialties or populations. We did not contact authors for clarification on unclear areas of RoB. While Cochrane is piloting a 2.0 bias assessment tool promising enhanced judgements, its core criteria remain largely unchanged. The introduction of a recent update to CONSORT in 2020 also further reinforces the importance of this item within avoiding uncertainty in reporting. Future research could consider using this updated tool.^36^

## Conclusion

The proportion of trials with inadequate methods and poor reporting within the field of IBD has reduced over time and in keeping with those of other specialties in the literature. However, almost half of the studies from the past 3 years carry potential bias risks, raising concerns for readers and researchers. Given GRADE’s widespread use in guideline development, these inconsistencies undermine confidence in IBD decision-making, highlighting the need for rigorous bias risk reporting among researchers and reviewers.

## Supplementary material

10.1136/bmjgast-2023-001337 online supplemental file 1

## Data Availability

Data are available upon reasonable request. All data relevant to the study are included in the article or uploaded as supplementary information.
