# Supplementary material for: Quality of reporting inflammatory bowel disease randomised controlled trials: a systematic review
Source: BMJ Open Gastroenterol. 2024 Apr 17;11(1):e001337. doi: 10.1136/bmjgast-2023-001337 (PMC11033348; doi:10.1136/bmjgast-2023-001337)
Supplement: online supplemental file 1 [file bmjgast-2023-001337supp001.pdf]

Appendix 1

Search strategy

(random[Title/Abstract] OR factorial[Title/Abstract] OR crossover[Title/Abstract] OR cross over[Title/Abstract] OR cross-over[Title/Abstract] OR placebo[Title/Abstract] OR single blind[Title/Abstract] OR double blind[Title/Abstract] OR triple blind[Title/Abstract] OR assign[Title/Abstract] OR allocate[Title/Abstract] OR randomized controlled trial[Title/Abstract]) AND (Crohn disease[Title/Abstract] OR Crohn*[Title/Abstract] OR Ulcerative colitis[Title/Abstract] OR ulcerative col*[Title/Abstract] OR IBD[Title/Abstract] OR Inflammatory bowel disease*[Title/Abstract])

Appendix 2

Analytical strategy

SPSS descriptive statistics and Chi-squared tests were conducted for statistical differences between pre-determined factors including studies published within journals with an IF to those with no IF, studies published in journals with an IF <5 to those with an IF >5, studies published in journals with an IF between 5-10 to those with an IF >10; studies with no-specified funding source to those with one, studies funded by public sponsorship to those funded by the industry, studies funded by the industry to those funded by both the industry and public sponsorship; studies published within the last three years to those published prior; and studies published by author groups with less than five authors to those with greater than five authors

Appendix 3 *Risk of Bias comparing study centre setting*

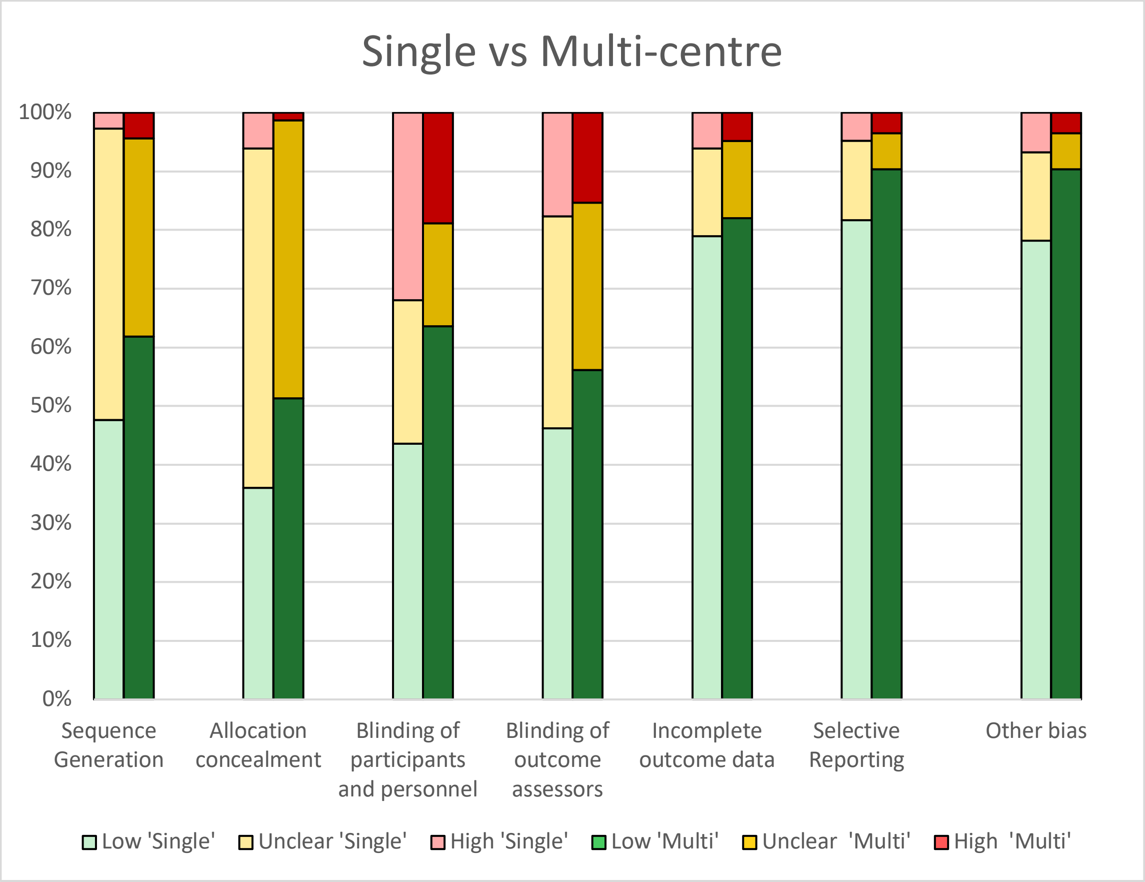

__Appendix 4__ Unclear risk of bias and progression of reporting over time

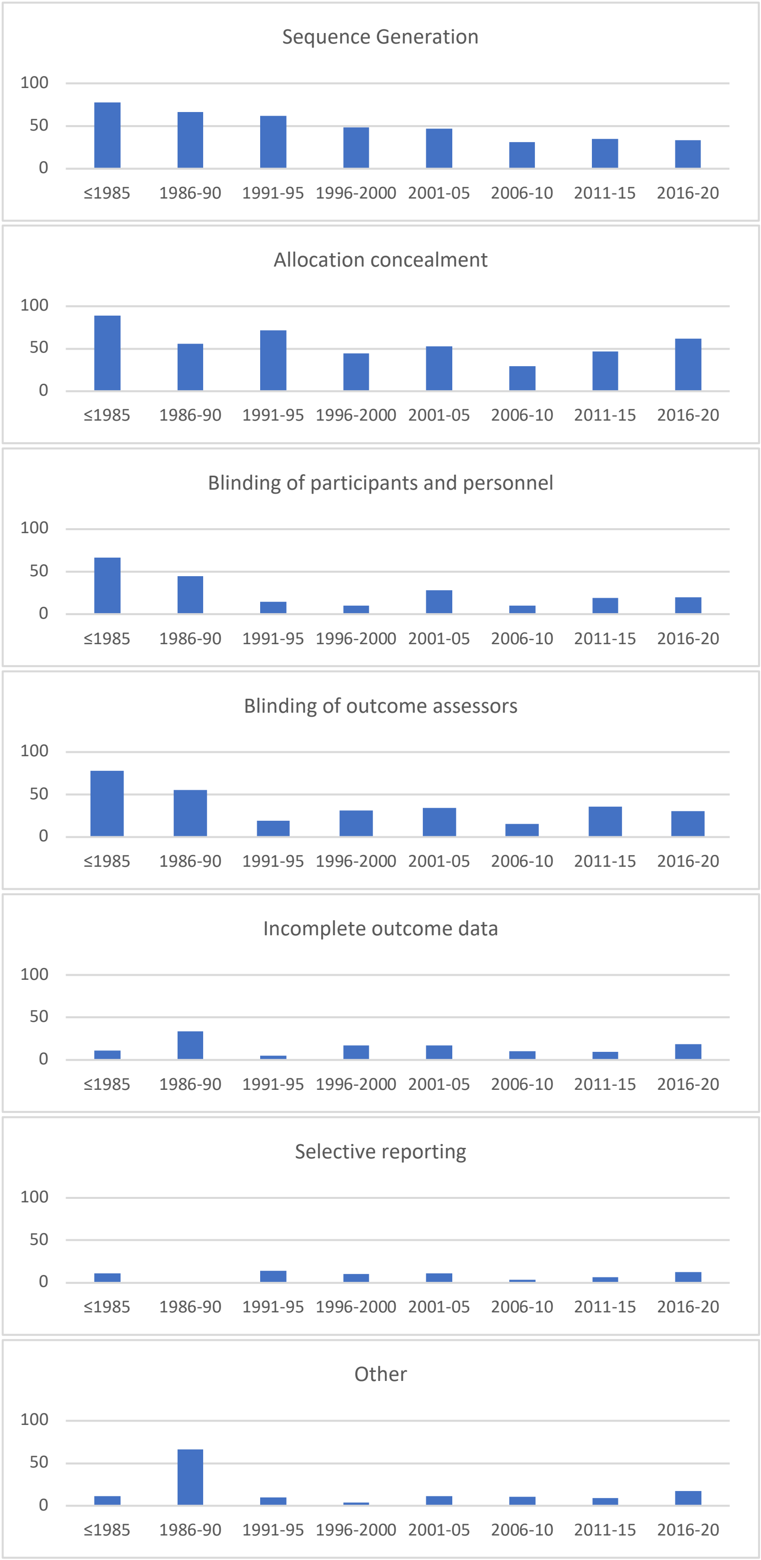

Appendix 5

Chi-squared comparison between pre-determined different factors

Impact Factor	SG for NIF to IF	*0.0122	SG for IF <5 to >5	**0.0019	SG for IF 5-10 to >10	0.7779
	AC for NIF to IF	0.0576	AC for IF <5 to >5	*0.0380	AC for IF 5-10 to >10	0.2957
	BPP for NIF to IF	0.1550	BPP for IF <5 to >5	*0.0284	BPP for IF 5-10 to >10	0.4271
	BOA for NIF to IF	*0.0228	BOA for IF <5 to >5	0.1252	BOA for IF 5-10 to >10	0.8664
	IOD for NIF to IF	*0.0241	IOD for IF <5 to >5	0.4051	IOD for IF 5-10 to >10	0.6523
	SR for NIF to IF	**0.0018	SR for IF <5 to >5	*0.0376	SR for IF 5-10 to >10	0.2218
	OR for NIF to IF	*0.0104	OR for IF <5 to >5	0.6130	OR for IF 5-10 to >10	0.8053
	p-value when comparing papers published in journals with different impact factors					

Funding	SG for NS to Funded	0.0583	SG for Public to Industry	0.2278	SG for Industry to I+P	0.9709
	AC for NS to Funded	*0.0029	AC for Public to Industry	0.8529	AC for Industry to I+P	0.1559
	BPP for NS to Funded	*0.0034	BPP for Public to Industry	0.0502	BPP for Industry to I+P	0.1576
	BOA for NS to Funded	0.0644	BOA for Public to Industry	*0.0187	BOA for Industry to I+P	0.6194
	IOD for NS to Funded	0.8951	IOD for Public to Industry	*0.0425	IOD for Industry to I+P	0.4394
	SR for NS to Funded	0.1222	SR for Public to Industry	*0.0316	SR for Industry to I+P	0.9260
	OR for NS to Funded	0.3804	OR for Public to Industry	0.0574	OR for Industry to I+P	0.4980
	p-value comparing papers with different funding sources					

Reporting over time	SG for <2017 to >2017	0.1152
	AC for <2017 to >2017	*0.0372
	BPP for <2017 to >2017	0.1557
	BOA for <2017 to >2017	0.0866
	IOD for <2017 to >2017	0.9310
	SR for <2017 to >2017	0.8664
	OR for <2017 to >2017	0.4742
	p-value when comparing papers published in the past 3-years to those previous	

Author group	SG for <5 to >5	*0.0140
	AC for <5 to >5	0.1544
	BPP for <5 to >5	*0.0206
	BOA for <5 to >5	0.1222
	IOD for <5 to >5	0.5509
	SR for <5 to >5	0.7845
	OR for <5 to >5	0.2338
	p-value when comparing papers published by different author group sizes	

Legend

SG - Sequence generation
AC - Allocation concelalment
BPP - Blinding of participants and personnel
BOA - Blinding of outcome assessors
IOD - Incomplete outcome data
SR - Selective reporting
OR - Other bias
IF - Impact factor
NIF - No IF
NS - Not specified
I+P - Industry & Public
* - p <0.05
** - p <0.01
